# Methodological and Practical Considerations Associated With Assessment of Alpine Skiing Performance Using Global Navigation Satellite Systems

**DOI:** 10.3389/fspor.2019.00074

**Published:** 2020-01-22

**Authors:** Matej Supej, Jörg Spörri, Hans-Christer Holmberg

**Affiliations:** ^1^Faculty of Sport, University of Ljubljana, Ljubljana, Slovenia; ^2^Department of Orthopedics, Balgrist University Hospital, University of Zurich, Zurich, Switzerland; ^3^Swedish Winter Sports Research Center, Mid Sweden University, Östersund, Sweden; ^4^China Institute of Sport and Health Science, Beijing Sport University, Beijing, China

**Keywords:** Galileo, GPS, GLONASS, biomechanics, trajectory, speed, velocity, energy dissipation

## Abstract

Reliable assessment of the performance of alpine skiers is essential. Previous studies have highlighted the potential of Global Navigation Satellite Systems (GNSS) for evaluating this performance. Accordingly, the present perspective summarizes published research concerning methodological and practical aspects of the assessment of alpine skiing performance by GNSS. Methodologically, in connection with trajectory analysis, a resolution of 1–10 cm, which can be achieved with the most advanced GNSS systems, has proven to provide acceptable accuracy. The antenna should be positioned to follow the trajectory of the skier's center-of-mass (CoM) as closely as possible and estimation of this trajectory can be further improved by applying advanced modeling and/or other computerized approaches. From a practical point of view, effective assessment requires consideration of numerous parameters related to performance, including gate-to-gate times, trajectory, speed, and energy dissipation. For an analysis that is both more comprehensive and more easily accessible to coaches/athletes, video filming should be synchronized with the GNSS data. In summary, recent advances in GNSS technology already allow, at least to some extent, precise biomechanical analysis of performance over an entire alpine skiing race course in real-time. Such feedback has both facilitated and improved the work of coaches. Thus, athletes and coaches are becoming more and more aware of the advantages of analyzing alpine skiing performance by GNSS in combination with advanced computer software, paving the way for the digital revolution in both the applied research on and practice of this sport.

## Introduction

In outdoor sports such as alpine skiing, valid and reliable assessment of performance in the field is essential, but, at the same time, quite challenging. For finding answers to many questions concerning performance, experiments under representative real-life conditions are indispensable and standardized procedures employing the latest advances in wearable technologies are central in this context. Previous studies have highlighted the considerable potential of Global Navigation Satellite Systems (GNSS) for evaluating alpine skiing performance based on monitoring of parameters such as time, speed and mechanical energy (Brodie et al., 2008b; Supej, 2010; Supej and Holmberg, 2011; Supej et al., 2013; Gilgien et al., 2014a, 2015a,b, 2016; Fasel et al., 2016; Kröll et al., 2016). The application of such technologies should allow both researchers and coaches to analyze the patterns of motion/tactics of athletes in depth and test sports equipment thoroughly.

Although alpine skiing performance can be characterized in terms of a variety of different biomechanical parameters, skiing from the start to the finish line as rapidly as possible is the obvious ultimate goal set by the International Ski Federation (FIS, 2016). Performance can be improved in many ways, but a skier can spend years attempting to gain a few hundredths of a second, which often make the difference between winning an Olympic medal or not. Nonetheless, the time spent on shorter sections of an alpine racing course can vary as much as 10%, even among the fastest skiers (Supej and Cernigoj, 2006), so there is considerable room for improving sectional performance.

Continuous adaptation of turning technique to changes in terrain, slopes, gate setup, and snow conditions is technically complex and requires assessment of parameters of performance that are more detailed and nuanced than simple overall race time (Supej, 2008; Supej et al., 2011; Federolf, 2012; Spörri et al., 2018). However, such analyses are extremely challenging, since many kinematic and kinetic factors influence performance, directly or indirectly. The most obvious kinematic parameters include section time, determined by the skier's trajectory and velocity; while the kinetic parameters include aerodynamics, interaction between the skis and snow (Federolf, 2012; Hébert-Losier et al., 2014), and energy dissipation, this latter constituting the best indicator of both instantaneous and sectional performance (Supej, 2008; Supej et al., 2011; Spörri et al., 2018). At present, no analytical approach provides a straightforward explanation of the difference between the fastest and slowest turns (Spörri et al., 2012). Clearly, multiple parameters must be monitored simultaneously.

Assessment of appropriate biomechanical parameters with modern wearable technology (such as the GNSS) can reveal minor, but nonetheless essential details concerning alpine skiing performance. Importantly, GNSS technology provides data quite rapidly, feasibly in real-time. However, insufficient accuracy and/or invalid methodology may lead to incorrect conclusions and reliable methodological advice concerning techniques, tactics and equipment is obviously a necessity. Accordingly, this commentary focuses on published research concerning methodological and practical considerations associated with the assessment of alpine skiing performance by GNSS.

## Practical Considerations

The multitude of biomechanical factors reported to influence performance include sectional or overall times, trajectory of the skis and/or of the center of mass, energy, ground reaction forces, aerodynamic drag, and frictional forces (Hébert-Losier et al., 2014). GNSS technology disturbs the skiers very little and is relatively easy to install and, therefore, has been applied extensively in assessing the performance of alpine skiers.

### Kinematic Parameters of Performance

In connection with competitive alpine skiing, accurate determination of section time is essential (Supej and Holmberg, 2011). To achieve the shortest overall time on a course, the skier should (1) lose as little time as possible on his/her weakest sections and gain as much as possible on the strong sections or (2) strive for his/her best time on each and every section (Supej and Cernigoj, 2006; Hébert-Losier et al., 2014). By determining gate positions and the skier's trajectory, GNSS technology enables assessment of gate-to-gate time with pronounced accuracy and validity (as compared to photocells) and no systematic bias, as well as an error that declines at higher velocities (Supej and Holmberg, 2011). Furthermore, multiple comparisons of gate-to-gate and lag times have demonstrated that GNSS enables much more detailed analysis than routine usage of photocells.

However, evaluation of performance on the basis of racing time alone, even on short sections of a course, involves several limitations (Supej, 2008). First, this time is influenced by the skier's initial velocity, position, and orientation. Secondly, the position and orientation at the end of a section relative to the following gate, as well as the exit speed will exert little influence on section time, but may affect subsequent performance profoundly. Third, such chronological information about when the skier is present at the initial and final positions of a section cannot explain why one skier is faster than another. Accordingly, other measures of performance are required.

In addition to racing time, skiing trajectory can also be analyzed by GNSS. In general, skiing the shortest possible trajectory rapidly results in the fastest time, but is often connected with higher energy losses (Supej, 2008; Federolf, 2012; Spörri et al., 2018). The ability to maintain high speed depends not only on the trajectory, but also on the skier's technique and choice of trajectory. Accordingly, valuable insights that improve performance can be gained by monitoring gate-to-gate times and velocities in order to analyze parameters related to skiing trajectory, such as whether the turn is started/ended at a higher or lower position in relationship to the gate or whether the trajectory is direct or more wider/rounded (Brodie et al., 2008a; Supej, 2008, 2012; Federolf, 2012; Spörri et al., 2012, 2018). This approach can also be of considerable value in connection with testing equipment (e.g., skis, ski boots), which in a broader sense is closely related to performance.

### Kinetic Parameters of Performance

Aerodynamic drag and ski–snow friction are the only two mechanical forces that can exert a detrimental impact on skiing performance (von Hertzen et al., 1997; Federolf et al., 2008; Meyer et al., 2012; Supej et al., 2013). When skiing downhill, aerodynamic drag accounts for almost 50% of the differences in racing time between slower and faster alpine skiers (Luethi and Denoth, 1987); whereas in the case of giant slalom, this drag causes only 15% of the total energy loss per turn and is not considered a major determinant of performance (Supej et al., 2013). The aerodynamic drag becomes more important as the speed increases (e.g., from slalom to downhill) (Gilgien et al., 2013, 2018). The opposite is true for ski-snow friction, which is more important at slower speeds, particularly when turning. During slalom and giant slalom, the ski-snow friction dissipates most of the energy (Supej et al., 2013). Even in the speed disciplines, involving more intense turning, the skiers focus more on guiding the skis smoothly than minimizing the frontal area exposed.

GNSS technology has been successfully applied to characterize aerodynamic drag (Gilgien et al., 2013; Supej et al., 2013) and ski-snow friction (Gilgien et al., 2013), as well as to estimate ground reaction forces during giant slalom, super-G, and downhill (Gilgien et al., 2014a). However, although this technology in combination with advanced computation and modeling, including double differentiation, is particularly suitable for investigating large differences between disciplines, such methodology is probably less able to detect small differences in performance.

Since aerodynamic drag makes only a small contribution to overall energy dissipation in the technical disciplines (giant slalom and slalom), it appears advisable to utilize overall energy dissipation, which is less prone to computational error, to assess performance in these cases (Supej, 2008; Supej et al., 2011). Moreover, as already discussed above, timing over a short section depends strongly on the performance in the preceding section (Supej et al., 2011) and the speed itself may change due to changes in, e.g., inclination. Therefore, analysis of energy, a more integral kinetic parameter, can improve evaluation of performance.

To summarize, no individual biomechanical parameter can explain *why* one skier is faster than another (Hébert-Losier et al., 2014). Kinematic parameters reflect more the outcome of performance (i.e., without consideration of cause), whereas kinetic parameters may provide insight into the underlying causes. Thus, successful skiers need to exploit the intricate interactions between biomechanical parameters and technique under varying conditions in a manner that minimizes section and/or overall race times.

## Methodological Considerations

### Validity and Reliability

GNSS in combination with integrated accelerometers has been applied extensively to a variety of sports during the past decade, e.g., for measuring the running velocity of team-sport players, both during matches and training (Johnston et al., 2012, 2014; Varley et al., 2012, 2014; Hoppe et al., 2018). Moreover, numerous investigations have focused on the validity and reliability of such systems for determining acceleration and/or speed during various forms of locomotion (Schutz and Herren, 2000; Townshend et al., 2008; Barbero-Alvarez et al., 2010; Coutts and Duffield, 2010; Aughey, 2011; Waldron et al., 2011; Varley et al., 2012; Akenhead et al., 2014; Johnston et al., 2014; Scott et al., 2016; Nagahara et al., 2017; Roe et al., 2017; Hoppe et al., 2018), utilizing photocells as the “golden standard” in most cases. Unfortunately, most such studies have not assessed velocities of relevance to competitive alpine skiing.

In the case of running, the validity and reliability of commercially available GNSS systems, even those of the same brand, is influenced by a number of factors. The slower the sample rate (Coutts and Duffield, 2010; Aughey, 2011; Varley et al., 2012), higher the velocity (Petersen et al., 2009; Jennings et al., 2010; Johnston et al., 2012), shorter the duration of activity (Petersen et al., 2009; Coutts and Duffield, 2010) and greater the number of changes in direction (Duffield et al., 2010; Jennings et al., 2010), the lower the validity and reliability of GNSS data. For example, a reduction in sampling frequency from 10 to 5 Hz can elevate both the standard error and coefficient of variation two- to three-fold (Waldron et al., 2011). On the other hand, even inexpensive GNSS systems measure speed with considerable accuracy, especially at lower acceleration (Supej and Cuk, 2014). Furthermore, real-time assessments with GNSS and/or synchronization of this technology with others (e.g., Inertial Motion Unit; IMU) requires caution, because of the pronounced latency of inexpensive systems, particularly those sampling at 1 Hz. In this context, it should be noted that the systems employed should be selected carefully, since some products advertise higher sampling rates that provide little or no benefit in practice (Haugen and Buchheit, 2016). On the other hand, a previous study proposed that supplementing GNSS with inexpensive and light-weight accelerometers offers a cheap and promising alternative with which to improve sampling rates and accuracy (Waegli et al., 2009).

At the same time, the validity and reliability of GNSS can be improved by employing high-quality differential/real-time kinematics (RTK) systems (Gilgien et al., 2014b), as is more often done in investigations on alpine skiing (Supej et al., 2008, 2013; Supej, 2010; Supej and Holmberg, 2011; Gilgien et al., 2013, 2015a,b, 2016, 2018). In fact, with their highest accuracy and applicability, RTK GNSS systems represent the “golden standard” for alpine skiing. In most studies on alpine skiing, an acceptable accuracy for trajectory analysis has proven to be 1–10 cm, at least when examining the technical disciplines (slalom and giant slalom), where gate distances and differences in trajectory are smaller than in the speed disciplines. Such great accuracy can only be achieved with differential, GPS+GLONASS (the United States plus the Russian global navigation satellite systems) and dual frequency GNSS systems (Gilgien et al., 2014b), working either in real-time (RTK) or post-processing kinematic mode. Improvements in the sensitivity of the receivers, an increase in the number of satellites, and development of tracking on multiple frequencies (e.g., the Galileo GNSS has four) are enhancing the position accuracy of newer, less expensive systems (GalileoGNSS, 2018).

Most high-quality RTK systems provide sampling rates of 10–20 Hz, sometimes as high as 100 Hz; however, based on our own experience, at such high frequencies accuracy is often compromised. In addition, even with optimal satellite visibility and accuracy on the order of 1 centimeter, monitoring of position at 100 Hz results in a high noise-to-signal ratio^1^ when determining, e.g., speed or, even worse, acceleration by double differentiation. This noise then needs to be filtered out by proper algorithms, most often using restrictive low-pass digital filters, which render such high sampling frequencies of questionable value.

Finally, to ensure optimal precision, the use of geometric dilution of precision (GDOP) and visible satellites with a sufficiently broad azimuth angle is advisable (Supej and Holmberg, 2011), since measurements are affected by the constantly changing constellation of the satellites involved (Parkinson and Spilker, 1996). Furthermore, during analysis of performance, the error for each position on the trajectory surveyed must be determined, since with GNSS technology this error may change rapidly.

### Positioning Antenna in Relationship to the Skier's Center-of-Mass

The major drawback of a single GNSS unit is that only a single trajectory, i.e., the path of the antenna, can be monitored. Obviously, placing the antenna on the skier's center-of-mass (CoM), from a biomechanical perspective probably the most interesting position to track, is quite difficult. Most commonly, the antenna is positioned either on the neck (in the vicinity of the upper thoracic spine) (Supej et al., 2008, 2013; Supej, 2010, 2012; Supej and Holmberg, 2011; Nemec et al., 2014) or on top of the head (typically on the helmet) (Gilgien et al., 2013, 2015a,b, 2016, 2018). Each of these placements has certain advantages and disadvantages: the antenna on the neck is closer to the CoM, but satellite visibility may be restricted. In contrast, placement on the head usually provides better satellite visibility, but the antenna is then further from the CoM, with a longer lever, while also restricting head movement and elevating the torque on the skier's neck in connection with rapid acceleration during turning, landing or similar actions. Placing multiple antennae on the skier in order to monitor the position of the CoM more accurately is not advisable, because this will reduce satellite visibility in the case of antennae positioned below the shoulders and wearable systems that are sufficiently accurate (e.g., RTK GNSS) are also bulky. Therefore, placement of the antenna on the skis, as suggested by Seifriz et al. (2003), is, in our opinion, also not desirable, since this may in addition also interfere with the behavior of the skis.

To improve analysis of the kinematics of the CoM on the basis of positioning data provided by GNSS antennae, various approaches have been explored. Supej (2008) modeled the skier's body as a statically balanced inverted pendulum and then employed Kalman filtering to estimate the acceleration required to compute the equilibrium position of this pendulum. A similar calculation of the quasi-static equilibrium of an inverted pendulum has been proposed by Gilgien et al. (2015b). In addition, in order to estimate the COM while taking into consideration the influence of air drag during giant slalom skiing, this approach has been improved further by taking the dynamics of the inverted pendulum into account as well (Supej et al., 2013).

In comparison to video-based 3D kinematics (Gilgien et al., 2013, 2015b) and inertial systems (Nemec et al., 2014), such inverted pendulum models provide accurate results during the more rapid turning in the vicinity of gates, where radial forces are stronger, but poorer results during weight transitions, which involve weaker forces. Novel solutions to this problem that have been proposed include locally weighted projection regression and back-propagation neural networks designed to predict the skier's posture from the GNSS data (Nemec et al., 2014), both of which demonstrate errors of prediction substantially smaller than those obtained with inverted pendulum models. Moreover, these novel approaches are computationally less demanding, allowing utilization in real-time.

Alternatively, multiple inertial sensors in combination with GNSS can be used to monitor whole-body 3D kinematics during alpine skiing (Brodie et al., 2008a; Krüger and Edelmann-Nusser, 2010; Supej, 2010), enabling detailed analysis over the entire course. Accordingly, when the movement of fewer body segments or only of the CoM is of interest, the number of inertial sensors can be reduced (Fasel et al., 2016). Although usage of multiple inertial sensors in combination with GNSS is of considerable value in connection with advanced scientific research, its methodological complexity makes it impractical for monitoring performance in the field. Nevertheless, adding an accelerometer to assist RTK GNSS may also be used to help to navigate zones where GNSS signals are unreliable (Skaloud and Limpach, 2003).

### Synchronizing GNSS With Video Recordings

Despite the many advantages of wearable GNSS technology, this technology alone does not provide feedback that is fully intuitive to athletes and their coaches. A relatively simple way to circumvent this difficulty is to combine video recordings with the GNSS. Qualitative visual information can greatly assist in the analysis of performance, especially since athletes and coaches routinely examine video recordings. For more advanced analysis, synchronization between the GNSS and video recording is necessary and this can be achieved with different types of hardware or even through simple body movements, such as squats (Supej, 2012).

## Future Perspectives

Again, it is worth noting that the differences in the race times of Olympic alpine skiers who take gold and silver medals are no more than hundredths of a second (e.g., 0.01 s in the case of the women's Super-G in the 2018 Pyeongchang Winter Olympics), rendering virtually all factors that influence performance extremely important. Although the technological ability to assess the biomechanics of alpine skiers has been improved substantially in recent decades, relatively little is yet known concerning optimization of performance over an entire course (Hébert-Losier et al., 2014) or about interactions between skiing on successive sections (Supej and Cernigoj, 2006). Recent advances in GNSS technology will and, to some extent, already do allow precise biomechanical analysis of performance over an entire race course in real-time (Supej et al., 2008, 2013; Supej, 2012; Gilgien et al., 2013), providing much more detailed information about such factors.

Continuous miniaturization of mechanical, electrical, and optical sensing technologies that enable assessment of the kinematics and kinetics of human motion and performance will lead to even more comfortable and flexible monitoring and assessment of the training load, technique, choice of trajectory and performance of alpine ski racers (Heikenfeld et al., 2018). In this connection, an innovative approach to estimating center-of-mass that involves fusing inertial sensors with anchor points that become available periodically has been proposed recently (Fasel et al., 2018). Further development of lighter and less bulky GNSS systems with accelerometers could provide a cheaper alternative to RTK systems in this context. More user-friendly and automated software involving artificial intelligence (machine learning, neural networks, and deep learning) in combination with wearable technology is expected to allow real-time feedback in the near future (Nemec et al., 2014). Therefore, coaches will need more technical and computer skills and/or expert assistance. Finally, in analyzing alpine skiing, both experts and coaches must become more aware of the possibilities offered by highly accurate GNSS.

## Conclusions

Wearable technologies, especially GNSS, have been widely used in research on alpine skiers. The biomechanical feedback provided by such technologies has improved and facilitated the work of coaches. Many skiing teams occasionally or even regularly employ GNSS technology to assess skiing performance and test equipment. This approach can help identify minor, but important differences between athletes that cannot be detected by the naked eye, standard photocells or video analysis (e.g., gate-to-gate timing, comparison of gate-gate velocities, and precise analysis of trajectory). Athletes and coaches are becoming more and more aware of the advantages of GNSS and other wearable technologies in combination with advanced computer software, paving the way for digital revolution of the science, as well as the practice of sports.

## Author Contributions

MS, JS, and H-CH contributed to all parts this paper, including the concept, design, and writing. All authors approved the final version for publication.

### Conflict of Interest

The authors declare that the research was conducted in the absence of any commercial or financial relationships that could be construed as a potential conflict of interest.

## Footnotes

^1^Monitoring two consecutive positions with a latitude accuracy of 1 cm and a sampling rate of 100 Hz is associated with a 2 m/s error in speed, which, after double differentiation, results in a 400 m/s^2^ error in acceleration. In contrast, the same position accuracy at 20 Hz is associated with corresponding errors of 0.4 m/s and 16 m/s^2^.
